# Brachial plexus injury after clavicle fracture operation: a case report and literature review

**DOI:** 10.1186/s12893-021-01335-8

**Published:** 2021-09-06

**Authors:** Zhenyu Cao, Yufei Hou, Xiaochen Su, Menghao Teng, Wenchen Ji, Meng Li

**Affiliations:** 1 Department of Orthopedics, First Affiliated Hospital of Xi’an Jiaotong University, Xi’an, 710061 China; 2 Department of Orthopedics, Qinghai Provincial People’s Hospital, Xining, 810007 China

**Keywords:** Clavicle fractures, Open reduction and internal plate fixation, Brachial plexus injury

## Abstract

**Background:**

Open reduction and internal fixation (ORIF) is the preferred choice for treating clavicle fractures. The brachial plexus injury caused by ORIF of a clavicle fracture is very rare. If it is not treated in time, the function of the brachial plexus will be challenging to recover, which will eventually lead to upper limb dysfunction and seriously affect the patient’s quality of life. Our team recently used ORIF to treat a patient with a clavicle fracture, who developed brachial plexus injury symptoms after surgery.

**Case presentation:**

A 34-year-old female patient was admitted to the hospital for 13 h due to the right shoulder movement restriction after a fall. Due to the significant displacement of the fracture, we used ORIF to treat the fracture. The surgery went well. When the anaesthesia effect subsided 12 h after the operation, the patient developed right brachial plexus injury symptoms, decreased right upper limb muscle strength, dysfunction, and hypoesthesia. Symptomatic treatments, such as nourishing nerve and electrical stimulation, were given immediately. Sixty days after the operation, the patient’s brachial plexus injury symptoms disappeared, and the function of the right upper limb returned to the preoperative state.

**Conclusions:**

Patients with clavicle fractures usually need to undergo a careful physical examination before surgery to determine whether symptoms of brachial plexus injury have occurred. Anaesthesia puncture requires ultrasound guidance to avoid direct damage to the brachial plexus. When the fracture end is sharp, reset should be careful to prevent nerve stump stabbed. When using an electric drill to drill holes, a depth limiter should be installed in advance to prevent the drill from damaging the subclavian nerve and blood vessels. When measuring the screw depth, the measuring instrument should be close to the bone surface and sink slowly to avoid intense hooks and damage to the brachial plexus. Try to avoid unipolar electrosurgical units to prevent heat conduction from damaging nerves, and bipolar electrocoagulation should be used instead. If symptoms of brachial plexus injury occur after surgery, initial symptomatic treatment is drugs and functional exercise, and if necessary, perform surgical exploration.

## Introduction

The shape of the clavicle is an S-shaped slender bone with a superficial position. It is prone to be fractured under the action of external forces. It accounts for 2–10% of all fractures in the body and 35% of scapular girdle fractures [1]. If not handled properly, it will often result in shoulder joint dysfunction. The most common cause of clavicle fractures is direct violence, such as car accident injuries, falls, sports injuries, etc; and can also be caused by indirect violence, such as the force of the palms on the ground when they fall [2, 3]. For fractures without apparent displacement, conservative treatment can often achieve good results, while for fractures with evident displacement, surgery is a better choice. ORIF is the preferred choice for treatment of clavicle fractures [4]. According to previous literature reports, some patients will experience complications such as fracture malunion, vascular injury, thoracic outlet syndrome, and pneumothorax, but brachial plexus injury is extremely rare [5–8]. Our team recently completed a case of a patient with a clavicle fracture, who developed symptoms of brachial plexus injury after surgery. The specific report is as follows:

## Case presentation

**Fig. 1:**
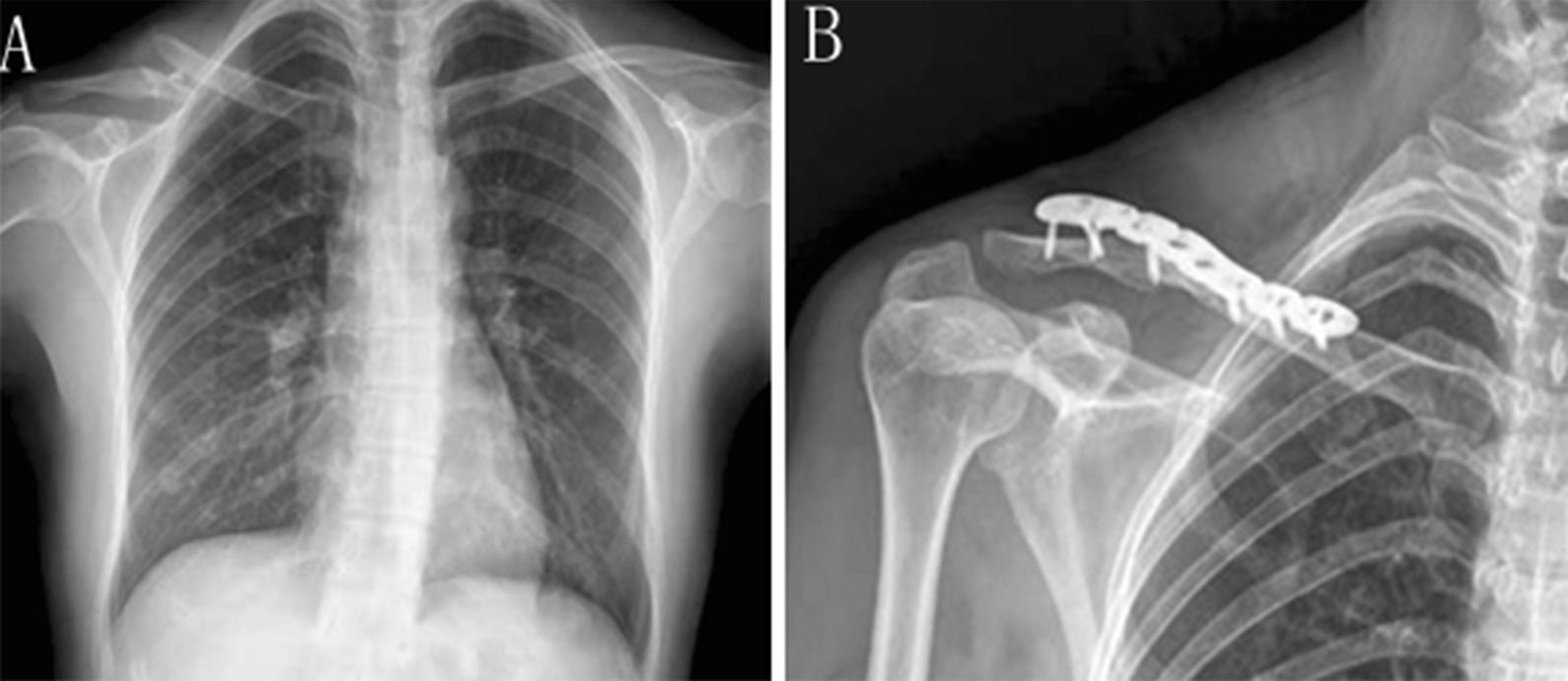
**A** Preoperative orthotopic chest radiograph. **B** Postoperative orthographic shoulder joint radiograph

A 34-year-old female patient was admitted to the hospital for 13 h due to the right shoulder movement restriction after a fall. The X-ray showed a fracture of the right clavicle (Fig. 1-A), with a significant displacement of the fracture end. After the fundamental assessment of the patient, we found that the patient’s upper limb nerve function is normal and can undergo the operation. So it was planned to use the clavicle anatomical plate (Waston Medical Corporation, China) for open reduction and internal fixation, following the standard operating procedure, using brachial plexus and cervical plexus anaesthesia, taking the fractured end as the center to make an 8 cm incision, and separating the tissue layer by layer. The fracture ends were severely comminuted, with the sharp distal and proximal ends. After carefully aligned the fracture ends with anatomical locking plates and screws, the fractures were well restored by radioscopy, and the wound is sutured after washing (Fig. 1-B).

**Fig. 2:**
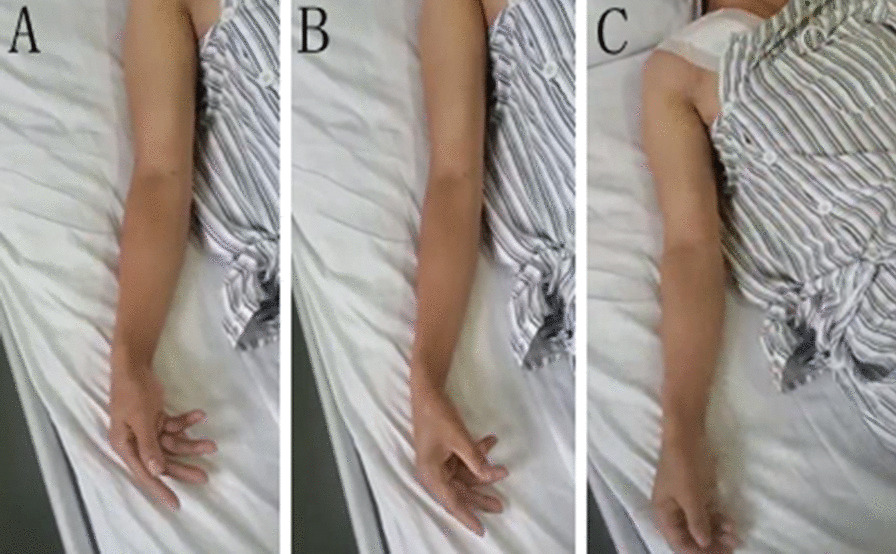
**A** Inability to extend the wrist. **B** Inability to extend the thumb. **C** Inability to flex the elbow

Twelve hours after the operation, the anesthetic effect subsided, and the patient complained of weakness in the right upper arm and forearm. Physical examination showed that the range of motion of the right elbow, right wrist joint, and right fingers was significantly reduced compared to the opposite side. The patient couldn’t extend the wrist (Fig. 2A), could extend the thumb (Fig. 2B), could flex the elbow (Fig. 2C). Muscle strength: right deltoid 2/5, right biceps 2/5, lateral extensor pollicis longus 2/5. The sensation of the right forearm and right hand was weaker than that of the contralateral side. Reports of brachial plexus injury caused by clavicle fracture surgery are rare but based on the patient’s symptoms, we consider the patient has incomplete brachial plexus injury. However, there is no indication for immediate surgical exploration because of the short occurrence time. Thus we gave supportive treatment: Oral methylcobalamin to nourish nerves (Yangtze River Pharmaceutical Group Corporation), 0.5 mg/time, 3 times/day, and limb nerve electrical stimulation to promote functional recovery, 20 min/time, 2 times/day. On the 3rd and 7th day after the operation, the patient’s surgical incision healed well. The range of motion of the right elbow, right wrist joint, and right thumb and index finger recovered slightly, but the range of motion of the 3rd, 4th, and 5th fingers of the right hand remained unchanged. There was no significant improvement in the muscle strength of the right deltoid, biceps, and right extensor pollicis longus.

**Fig. 3:**
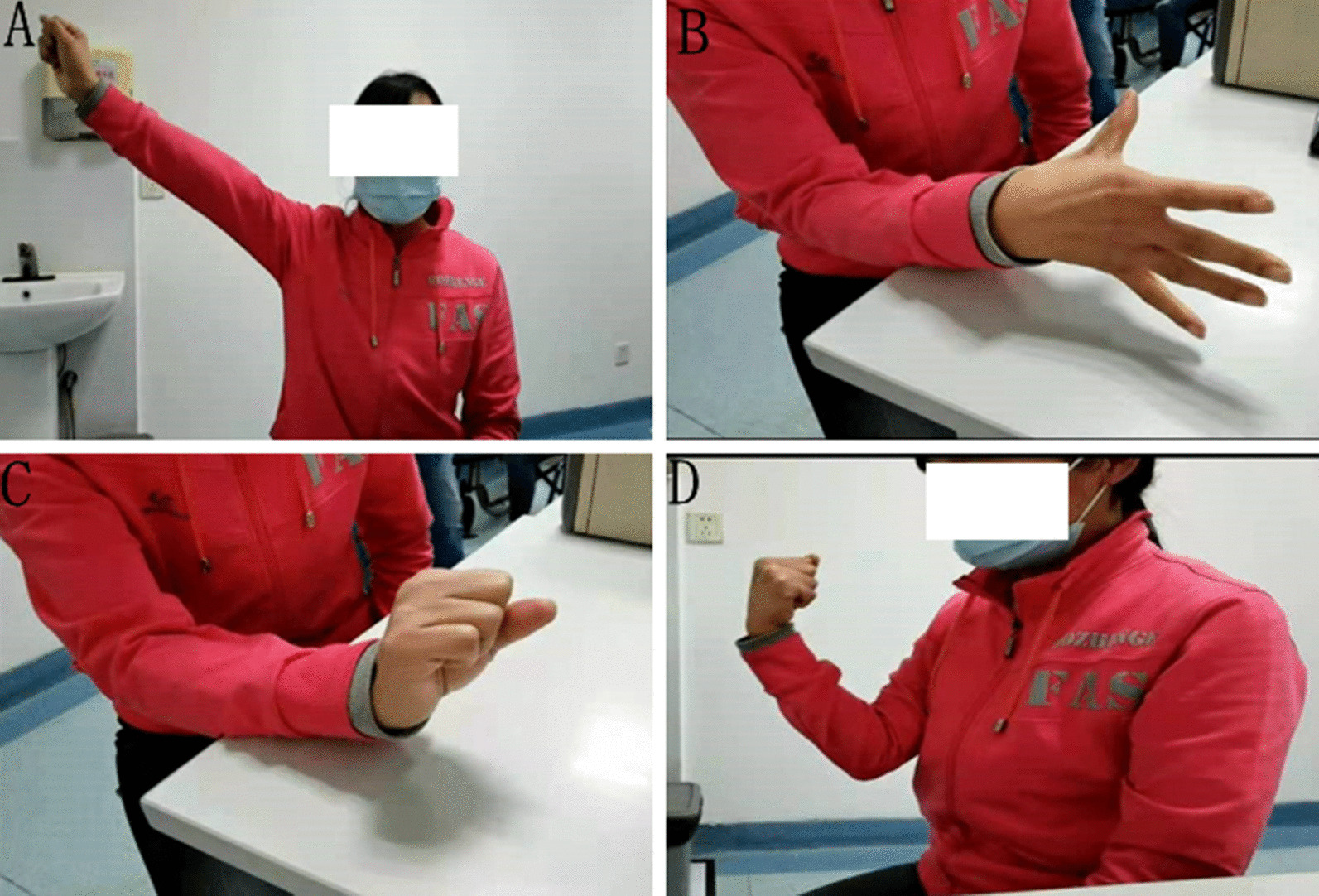
**A** Shoulder abduction. **B** Abduction and adduction of the fingers. **C** Wrist extension. **D** Elbow flexion

After being discharged from the hospital, the patient was asked to continue rehabilitation exercises and nutritional nerve treatment; and was followed every 2 weeks to 60 days after surgery. The motor function of the patient’s right upper limb recovered well. Abduction and adduction of shoulder and fingers, flexion and extension of wrist and elbow were normal (Fig. 3). Muscle strength: right upper limb completely restored, right deltoid 5/5, right biceps 5/5, right extensor pollicis longus 5/5. The sensation of the right forearm and right hand shows no abnormality compared to the opposite side.

## Discussion and conclusions

The brachial plexus is an important peripheral nerve plexus in the human body. It is composed of C5–C8, T1 nerve roots, and innervates the sensory and motor functions of the shoulder and upper limbs [9]. Once injured, the disability rate is high, which seriously affects the quality of life of patients. There are two most common causes of clavicle bone fracture with brachial plexus injury: one is in newborns with the injury caused by the traction of the newborn’s upper limb during the delivery; the other is in adults with the injury caused by traffic accidents, falling from heights, etc [10, 11]. The symptoms caused by puncturing a nerve at the fracture’s broken end often appear immediately after the injury [12]. According to previous reports, the probability of brachial plexus injury caused by clavicle fracture surgery is extremely rare.

The patient had no apparent symptoms of brachial plexus injury immediately after the injury. The surgical procedures are gentle overall. There are no procedures that result in hyperextension of the shoulder, hyperabduction of the upper limb. When surgery is completed, we give the careful hemostasis, and there is no active bleeding forming a partial hematoma which may compress the nerves. However, after the anesthesia wears off, the patient developed symptoms of brachial plexus injury, mainly manifested as loss of shoulder elevation due to deltoid muscle dysfunction innervated by axillary nerve, loss of elbow flexion due to biceps muscle dysfunction innervated by musculocutaneous nerve, loss of the sensory and movement of the corresponding area of the radial nerve, median nerve and ulnar nerve. Analyzing the causes of brachial plexus injury, it is believed that there are the following possibilities: (1) Ultrasound navigation is not used during the anesthesia, and the puncture needle may directly puncture the brachial plexus based on experience; (2) The fracture ends are sharper, which may scratch the nerves during the reduction process; (3) There is variation in the brachial plexus, which are close to the fractured end. Heat conduction can cause nerve damage when using an electric knife to separate soft tissues. Clinically, the essential methods for early diagnosis of brachial plexus injury are electromyography examination and magnetic resonance imaging (MRI), which have the advantages of rapid, non-invasive, and high accuracy [13, 14]. Ultrasound is also an alternative examination method, but it has great requirements for the operator, which is not suitable for primary hospitals [15].

However, it is generally recommended to be performed electromyography examination 3 weeks later after the surgery, for the early soft tissue edema around the wound affects the diagnostic accuracy. On the second day after the operation, we diagnosed the brachial plexus injury according to the physical examination and make a detailed recovery plan. Methylcobalamin and electrical stimulation are commonly used methods in clinic and basic research area, which plays an important role in the treatment of nerve injury and degeneration. Zhang demonstrate that methylcobalamin has the ability to facilitate the recovery of motor and sensory function, and to promote myelination in peripheral nerve regeneration [16]. Willand think the imminent clinical applicability of acute brief low-frequency electrical stimulation effectively promote axonal regeneration and maximize functional recovery in diverse types of peripheral nerve injuries [17]. So we gave oral methylcobalamin to nourish the affected limb’s nerves and daily electrical stimulation to promote functional recovery. It is planned that if there is no improvement after 3 weeks, perform the electromyography. MRI is a very good way to diagnose brachial plexus injury. However, this patient had just undergone internal fixation of the fracture, and immediately improved MRI may lead to fracture displacement and internal fixation failure. If there is no relief after 3 months, we will perform MRI and surgical exploration. With regular patient’s follow-up after discharge, the symptoms gradually improved, and disappeared completely recovered to the preoperative state by 60 days after the operation, so we did not have an MRI examination on him.

Through this patient, we advise that patients with clavicle fractures maybe need to concern the following points for ORIF in order to avoid brachial plexus injury: (1) Careful physical examination is required before surgery to determine whether brachial plexus injury symptoms have occurred, if it appears, we need to exploration of nerve during operation. (2) Using ultrasound-guided needle puncture as much as possible during the anesthesia puncture process to avoid direct damage to the brachial plexus. (3) When relatively sharp fracture fragments, the reduction maybe careful to prevent puncturing local nerve ends. (4) When using an electric drill, it is better to install a depth limiter in advance. It is generally safer to limit it to within 1.8 cm to prevent the drill from damaging the subclavian nerve and blood vessels. (5) When measuring the depth of the screw, the measuring instrument against the bone surface and sink slowly to avoid snagging brachial plexus, while gently inserted under the clavicle bone aside for added protection. (6) Try to avoid using a single-stage electric knife, in order to avoid heat conduction nerve damage. In particular, those whom some patients may have anatomic variation close to the fracture. Bipolar coagulation is recommended. (7) If the patient develops brachial plexus injury symptoms after surgery, the preferred supportive treatments are drugs and rehabilitation training such as electrical stimulation. Suppose the symptoms are not significantly relieved until 3 weeks after surgery. In that case, it is advised to perform the electromyography to identify the specific injury part, and if the nerve function still does not recover until 3 months after the operation, we need do MRI and surgical exploration is recommended.

## Data Availability

The datasets during and/or analyzed during the current study are available from the corresponding author on reasonable request.

## Abbreviation

ORIF: Open reduction and plate internal fixation
